# Efficacy and safety of cardiac myosin inhibitors for symptomatic hypertrophic cardiomyopathy: a meta-analysis of randomized controlled trials

**DOI:** 10.3389/fcvm.2024.1477487

**Published:** 2025-01-15

**Authors:** Anas Abunada, Madiha Shah, Ateesh Kumar, Syeda Lamiya Mir, Dinesh Kumar, Saboor Ahmed, Muhammad Tanzeel, Vikash Kumar, Aashish Meghjiani, Muhammad Basit Ali Siddiqui, Govinda Khatri, Aneesh Rai, Fnu Deepak, Ayush Kumar

**Affiliations:** ^1^ Department of Cardiology, Liaquat University of Medical & Health Sciences, Jamshoro, Pakistan; ^2^ Dow Medical College, Dow University of Health Sciences, Karachi, Pakistan; ^3^ Department of Cardiology, Shaheed Mohtarma Benazir Bhutto Medical College Lyari, Karachi, Pakistan; ^4^ Department of Cardiology, Jinnah Sindh Medical University, Karachi, Pakistan; ^5^ Department of Cardiology, Vayodha Hospitals, Kathmandu, Nepal

**Keywords:** hypertrophic cardiomyopathy, cardiac myosin inhibitors, mavacamten, aficamten, LVOT obstruction

## Abstract

**Introduction:**

Hypertrophic cardiomyopathy (HCM) is a common genetic heart disorder. It is characterized by left ventricular hypertrophy and impaired cardiac function, with forms categorized into obstructive (oHCM) and nonobstructive (nHCM). Traditional treatments address symptoms but not the underlying disease mechanism, highlighting the need for novel therapies. Cardiac myosin inhibitors such as mavacamten and aficamten present potential new treatment options.

**Methods:**

A meta-analysis of randomized controlled trials (RCTs) was conducted following PRISMA guidelines. Studies comparing cardiac myosin inhibitors with placebo were reviewed, and outcomes related to NYHA functional class, Kansas City Cardiomyopathy Questionnaire Clinical Summary Score (KCCQ-CSS), LVOT gradients, and left ventricular ejection fraction (LVEF) were analyzed.

**Results:**

Six RCTs involving 826 participants demonstrated that mavacamten and aficamten significantly improved NYHA functional class and KCCQ-CSS scores. The incidence of treatment-emergent adverse events (TEAEs) and serious adverse events (SAEs) was similar between the treatment and control groups, indicating a comparable safety profile.

**Conclusion:**

Cardiac myosin inhibitors are effective in improving cardiac function and reducing LVOT obstruction in HCM patients. They offer a promising alternative to current treatments, with a safety profile comparable to placebo. Further research is needed to confirm long-term benefits.

## Introduction

Hypertrophic cardiomyopathy (HCM) is the most common hereditary cardiovascular disorder in youth and adolescents. It is characterized by asymmetric left ventricular hypertrophy, especially at the basal interventricular septum, and affects the mitral and subvalvular regions, intracavitary space, and outflow tract. HCM exhibits myocardial disarray, microvascular pathology, decreased compliance, and cardiac fibrosis (1–3). As an autosomal dominant condition, its presentation varies due to incomplete penetrance and diverse sarcomere gene mutations (4). HCM is classified into obstructive (oHCM), with dynamic Left ventricular outflow tract (LVOT) obstruction, and nonobstructive (nHCM), which lacks significant LVOT obstruction (<30 mmHg) at rest or with provocation (5). LVOT obstruction can result in severe symptoms such as heart failure, atrial fibrillation, reduced exercise capacity, exertional syncope, and increased risk of sudden death from malignant arrhythmias (6, 7).

The management of symptomatic oHCM primarily involves beta-blockers and calcium channel blockers, which alleviate symptoms through negative inotropic and chronotropic effects. However, these medications do not address the underlying pathophysiology, highlighting the need for novel treatments (8). Septal reduction therapy, including surgical myectomy and alcohol septal ablation (ASA), is recommended for patients unresponsive to medical management. Myectomy has a 0.5% mortality rate and improves quality of life in over 90% of patients by at least one New York Heart Association (NYHA) class. ASA offers shorter hospital stays and recovery times but has higher rates of complete heart block and arrhythmia, with 10%–15% of patients requiring a permanent pacemaker compared to less than 5% for myectomy. This underscores the urgent need for long-term treatments targeting the root causes of the disease (8–10).

A novel class of pharmacologic agents, the cardiac myosin inhibitors, notably mavacamten (MYK-461) and aficamten (CK-274), has recently emerged as a therapeutic alternative for HCM. These agents function as reversible inhibitors of cardiac myosin, thereby diminishing left ventricular contractility by reducing the number of active actin–myosin cross-bridges within the sarcomere, which are implicated in the myocardial hypercontractility characteristic of HCM (11, 12). Mavacamten, having recently received approval, has demonstrated efficacy in enhancing exercise capacity and alleviating symptoms in patients with obstructive HCM (13). In contrast, aficamten has been engineered to exhibit a shallow dose-response relationship, characterized by minimal reductions in left ventricular ejection fraction with increasing dosages, thereby indicating a broad therapeutic window and possessing a plasma half-life that facilitates personalized dose adjustments, potentially as frequently as every 2 weeks (11).

To date, a mere six randomized controlled trials (RCTs) have assessed the efficacy and safety of cardiac myosin inhibitors in HCM patients compared to placebo (14–19). While these trials have yielded promising clinical outcomes, it remains uncertain whether these benefits will be replicated on a larger scale. This study aims to amalgamate and enhance the statistical power of existing data to produce a more precise summary estimate of the clinical impact of myosin inhibitors in HCM patients.

## Methods

This meta-analysis conformed to the PRISMA guidelines for systematic reviews and meta-analyses and was executed following the framework established by the Cochrane Collaboration (20, 21).

### Literature search

A comprehensive literature search was conducted across PubMed, Cochrane Central, ScienceDirect, and ClinicalTrials.gov databases from their inception until August 2024. This search was unrestricted by time, language, or sample size constraints. The strategy employed included Medical Subject Heading (MeSH) terms and keywords such as “cardiac myosin inhibitors,” “Mavacamten,” “MYK-461,” “Aficamten,” “CK-274,” “hypertrophic cardiomyopathy,” “symptomatic hypertrophic cardiomyopathy,” and “obstructive hypertrophic cardiomyopathy.” We meticulously examined study titles, abstracts, full texts, and bibliographies of all identified research. Our evaluation included a comprehensive review of references within relevant literature to identify potentially pertinent studies, without any restrictions regarding geographical location, ethnicity, or publication language.

### Data extraction

The systematic search yielded numerous articles, which were imported into EndNote Reference Manager (Version X7.5; Clarivate Analytics, Philadelphia, Pennsylvania). Within EndNote, we meticulously screened for and removed duplicate entries. Initially, two reviewers independently assessed the titles and abstracts of publications that met the inclusion criteria, followed by a thorough review of the full texts. Data from eligible trials were then extracted and recorded in an information-extraction table. Key information collected included the first author, year of publication, NCT number, sample size, participant sex and age, baseline characteristics, and follow-up period. Any discrepancies in article selection and data extraction were resolved through discussion or by consulting a third reviewer.

### Inclusion criteria and outcomes

#### Study inclusion criteria

This study adhered to rigorous eligibility criteria for research studies, which included the following parameters: (a) RCTs featuring at least one intervention group administered Mavacamten or Aficamten compared to a control group receiving a placebo; (b) adult patients (≥18 years) with symptomatic HCM, encompassing both obstructive and non-obstructive forms.

Studies were excluded for various reasons, such as unsuitable design (including non-randomization), lack of data relevance, involvement of animal models, or if they were case reports, editorials, reviews, conference abstracts, or duplicate publications.

#### Outcomes of interest

The primary endpoints of interest encompassed symptomatic enhancement, as evidenced by an improvement of at least one grade in the NYHA functional classification, and modifications in the Kansas City Cardiomyopathy Questionnaire's Clinical Summary Score (KCCQ CSS) from the baseline evaluation. Secondary endpoints included alterations in the Valsalva LVOT peak gradient, rest LVOT peak gradient, post-exercise LVOT gradient, and changes in left ventricular ejection fraction (LVEF). Safety was assessed by the incidence of treatment-emergent adverse events (TEAEs) and serious adverse events (SAEs).

### Risk of bias assessment

The risk of bias in each included RCT was meticulously scrutinized utilizing the RoB 2 tool. This instrument examines several categories, including the generation of random sequences, allocation concealment, blinding of participants and personnel, blinding of outcome assessment, incomplete outcome data, selective reporting, and other potential sources of bias. The risk of bias in each domain was systematically categorized as low, high, or unclear (22).

### Statistical analysis

All statistical computations were executed using the Review Manager 5.3 (RevMan 5.3) software. The Mantel-Haenszel method was utilized for dichotomous outcomes, with results reported as risk ratios (RRs) and their corresponding 95% confidence intervals (CIs). For continuous variables, the inverse variance method was employed to calculate the mean difference (MD) and its respective 95% CI. A random effects model was adopted to address potential heterogeneity among studies. Heterogeneity was assessed using Cochrane's Higgins *I*^2^ and *Q* statistics. The *I*^2^ statistic quantifies the proportion of variation across studies that is due to heterogeneity rather than chance or sampling error, with *I*^2^ values below 50% indicating low heterogeneity, values above 50% indicating moderate heterogeneity, and values of 75% or higher indicating substantial heterogeneity. Subgroup analysis was conducted based on the specific drugs administered in the studies. To identify and mitigate sources of substantial heterogeneity, sensitivity analyses using the leave-one-out method were planned. Statistical significance was determined with a stringent *p* value threshold of less than 0.05 (23).

## Results

### Study screening and selection

**Figure 1:**
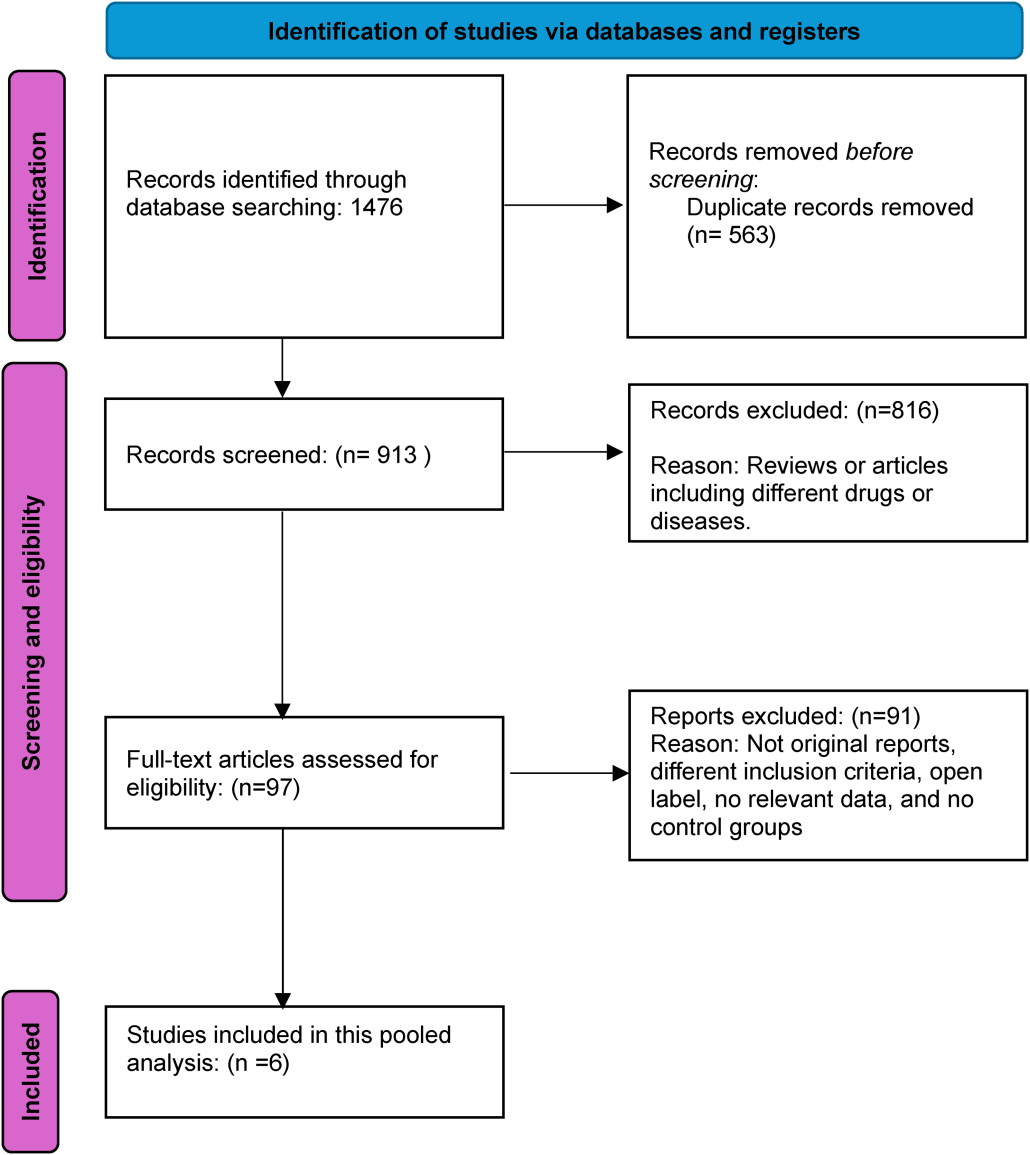
Prisma flow diagram.

The initial database search yielded 1,476 results, necessitating a systematic review to eliminate redundancies. After excising 563 duplicates, 913 studies remained, whose titles and abstracts underwent rigorous screening. This process excluded 816 citations irrelevant to the research focus. Subsequently, the full texts of 97 studies were meticulously reviewed for data on the intervention's safety and efficacy, resulting in the exclusion of 91 articles that did not meet the inclusion criteria. The final analysis incorporated 6 studies—2 comparing aficamten with placebo (14, 15) and 4 comparing mavacamten with placebo (16–19), meeting the inclusion criteria and providing significant insights into the research topic. Figure 1 illustrates the PRISMA flow diagram, detailing the study selection process.

### Baseline and study characteristics

This analysis encompassed six RCTs involving a total of 826 participants. Among these, 170 patients were administered aficamten, 273 received mavacamten, and 383 were assigned to the placebo group. The mean age of the participants spanned from 51 to 60.9 years. The demographic distribution was predominantly male, comprising 427 men and 399 women. Table 1 provides a detailed elucidation of the baseline characteristics and study features of the included trials.

**Table 1:** Study and baseline characteristics.

Study name, year and NCT number	Total population (*n*)	Groups	Participants (*n*)	Sex	Age	NYHA functional class (n)		KCCQ-CSS	Follow up	Dosing
				M/F	Mean (SD)	II	III	Mean (SD)		Oral
Maron et al. 2023 (14) NCT04219826	41 oHCM	Aficamten	14	10/4	55 (20.74)	10	4	-	10 weeks	14 with 5 mg aficamten
		Aficamten	14	3/11	60.66 (14.97)	7	7	-		
		Placebo	13	5/8	58.66 (8.14)	11	2	-		14 with aficamten 10 mg
Maron et al. 2024 (15) NCT05186818	282 oHCM	Aficamten	142	86/56	59.2 (12.6)	108	34	76 (18)	24 weeks	5 mg aficamten
		Placebo	140	81/59	59.0 (13.3)	106	33	74 (18)		
Desai et al. 2022 (16) NCT04349072	112 oHCM	Mavacamten	56	29/27	59.8 (14.2)	4	52	69.5 (16.3)	16 weeks	5 mg mavacamten
		Placebo	56	28/28	60.9 (10.5)	4	52	65.6 (19.9)		
Ho et al. 2020 (17) NCT03442764	59 nHCM	Mavacamten	40	19/21	54.0 (14.6)	33	7	-	16 weeks	5 mg mavacamten
		Placebo	19	6/13	53.8 (18.2)	13	6	-		
Olivotto et al. 2020 (19) NCT03470545	251 oHCM	Mavacamten	123	66/57	58·5 (12·2)	88	35	-	30 weeks	5 mg mavacamten
		Placebo	128	83/45	58·5 (11·8)	95	33	-		
Tian et al. 2023 (18) NCT05174416	81 oHCM	Mavacamten	54	41/13	52.4 (12.1)	44	10	82.4 (16.9)	30 weeks	5 mg mavacamten
		Placebo	27	17/10	51.0 (11.8)	18	9	84.4 (17.0)		

### Risk of bias assessment

**Figure 2:**
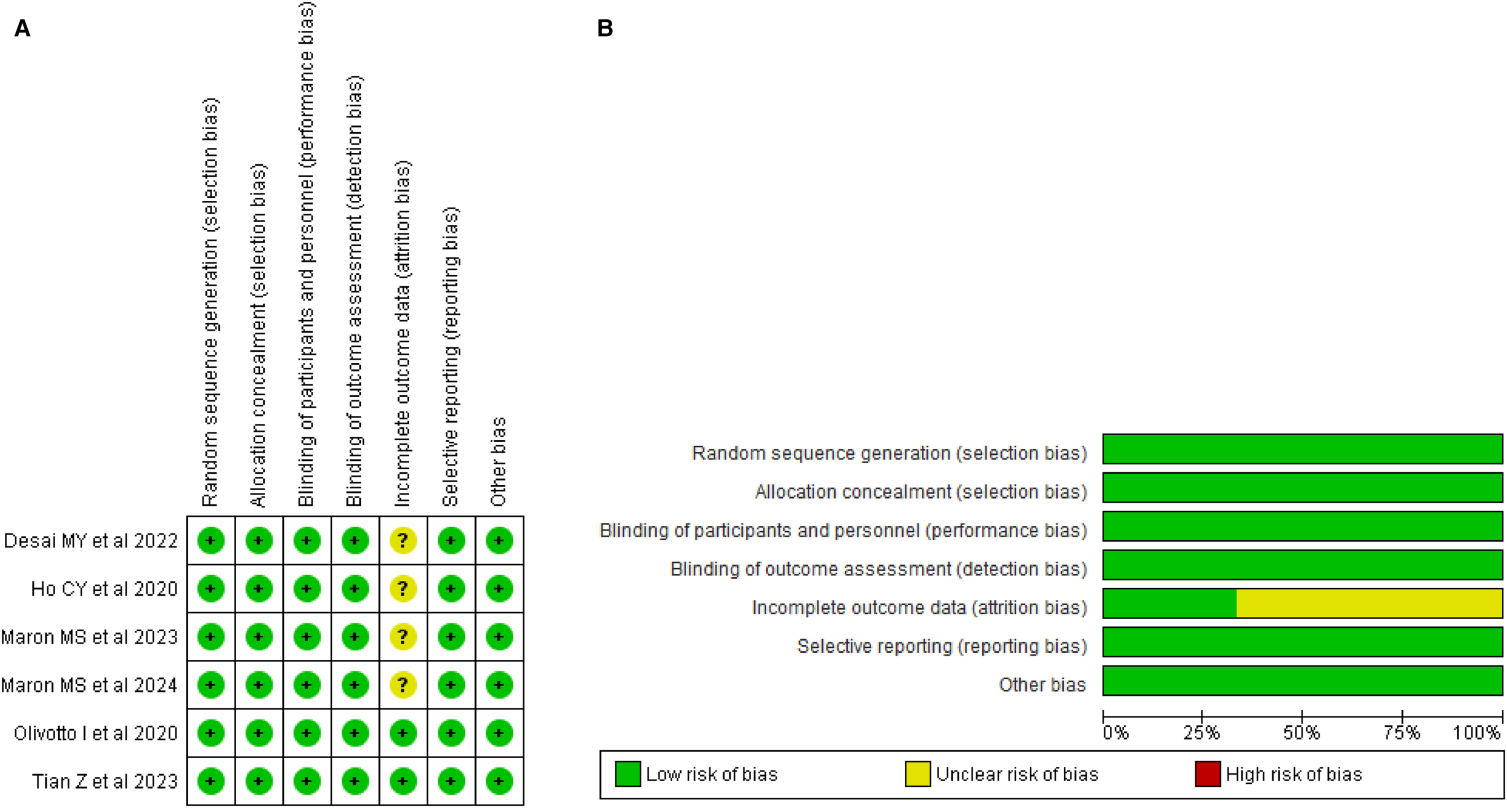
**(A)** Risk of bias summary. **(B)** Risk of bias graph.

The risk of bias assessment across the studies included in this review demonstrates a generally low risk of bias in several domains. Most studies employed random sequence generation and allocation concealment through methods such as interactive response systems, ensuring unbiased treatment allocation. Blinding of participants, personnel, and outcome assessors was consistently implemented, effectively minimizing performance and detection biases. However, several studies provided limited details on handling incomplete outcome data, leading to an unclear risk of attrition bias, particularly regarding missing data and dropouts. Overall, there is no evidence of selective reporting, and most trials adhered to good clinical practices with independent oversight, reducing the likelihood of other biases. The thorough assessment of these studies is illustrated in Figures 2A, 2B. Supplementary Table 1 contains detailed author judgments for each study, providing a comprehensive overview of the evaluation process.

### Meta-analysis

#### Improvement in NYHA functional class

**Figure 3:**
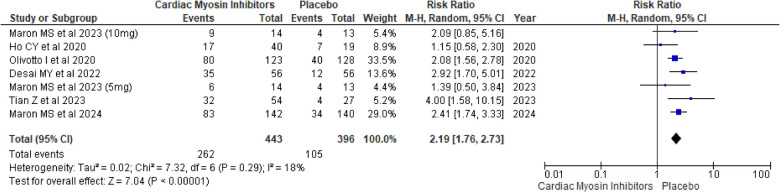
Forest plot for improvement in NYHA functional class.

The pooled analysis demonstrated a significant improvement in NYHA functional class for patients receiving the Aficamten/Mavacamten, with a RR of 2.19 (95% CI: 1.76, 2.73; *p* < 0.00001; *I*^2^ = 18%; Figure 3).

A subgroup analysis based on the drugs was conducted, and both drugs showed significant improvement in NYHA Functional Class in comparison to placebo, with aficamten showing a *p*-value of <0.00001 and mavacamten showing a *p*-value of 0.0001 (Supplementary Figure 1).

#### Improvement in KCCQ-CSS

**Figure 4:**
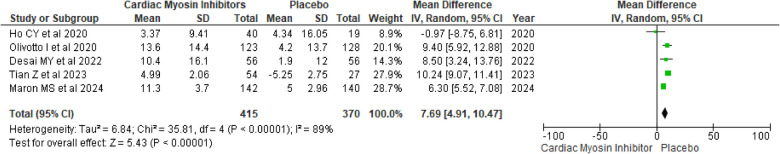
Forest plot for improvement in KCCQ-CSS.

The KCCQ-CSS, which measures patient-reported outcomes related to symptoms, physical limitations, and quality of life, showed a MD of 7.69 (95% CI: 4.91, 10.47; *p* < 0.00001; *I*^2^ = 89%; Figure 4). This significant improvement underscores the positive impact of the treatment on patients' perceived health status and daily functioning, although the high *I*^2^ value suggests substantial heterogeneity across the included studies.

A drug-based subgroup analysis revealed that both treatments significantly enhanced KCCQ-CSS compared to placebo, with each drug demonstrating a *p*-value of <0.00001 (see Supplementary Figure 2).

A sensitivity analysis was performed to mitigate heterogeneity by excluding a particular study, which substantially decreased variability and markedly enhanced the mean difference in KCCQ-CSS (MD = 6.80; *p* < 0.00001) (see Supplementary Figure 3) (18). Heterogeneity was reduced from 89% to 57% following this adjustment. This observed heterogeneity is likely attributable to the divergent baseline KCCQ-CSS scores in that single trial.

#### Mean Valsalva LVOT peak gradient

**Figure 5:**
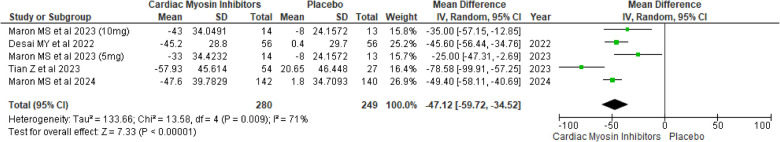
Forest plot for mean Valsalva LVOT peak gradient.

For the mean Valsalva LVOT peak gradient, the intervention group experienced a substantial reduction with a MD of −47.12 (95% CI: −59.72, −34.52; *p* < 0.00001; *I*^2^ = 71%; Figure 5).

A drug-specific subgroup analysis indicated that both Aficamten and Mavacamten significantly diminished the mean Valsalva LVOT gradient compared to placebo, with a *p*-value of <0.00001 0.0002 (see Supplementary Figure 4).

A sensitivity analysis was conducted to address heterogeneity by removing a particular study (18), which substantially reduced variability and significantly enhanced the mean difference in Mean Valsalva LVOT Peak Gradient (MD = −43.14; *p* < 0.00001) (see Supplementary Figure 5). This adjustment lowered heterogeneity from 71% to 38%. The observed heterogeneity was primarily attributed to the differing baseline Valsalva LVOT peak gradient scores in that single trial.

#### Mean rest LVOT peak gradient

**Figure 6:**
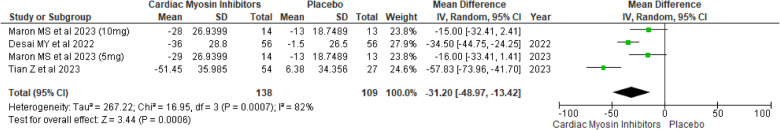
Forest plot for mean rest LVOT peak gradient.

The analysis revealed a significant reduction in the mean rest LVOT peak gradient, with a MD of −31.20 (95% CI: −48.97, −13.42; *p* = 0.0006; *I*^2^ = 82%; Figure 6). This reduction suggests that the treatment effectively decreases the LVOT gradient even at rest.

A drug-specific subgroup analysis revealed that both aficamten and mavacamten significantly reduced the mean Rest LVOT gradient compared to placebo, demonstrating a *p*-value of 0.01 and <0.0001 respectively (see Supplementary Figure 6).

A sensitivity analysis was undertaken to address heterogeneity by excluding a specific study (18), which slightly decreased variability from 82% to 63% (see Supplementary Figure 7).

#### Change in LVEF

**Figure 7:**
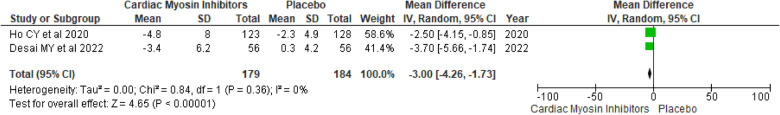
Forest plot for change in LVEF.

Changes in LVEF were also assessed, showing a modest but significant decrease with a MD of −3.0 (95% CI: −4.26, −1.73; *p* < 0.00001; *I*^2^ = 0%; Figure 7).

#### Post-exercise LVOT gradient

**Figure 8:**
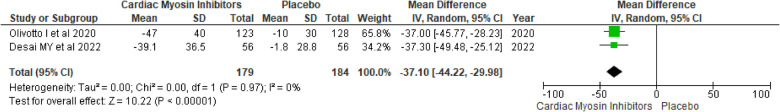
Forest plot for post-exercise LVOT gradient.

The post-exercise LVOT gradient showed a significant reduction with a MD of −37.10 (95% CI: −44.22, −29.98; *p* < 0.00001; *I*^2^ = 0%; Figure 8). This finding indicates that the Mavacamten effectively reduces LVOT obstruction even during physical exertion, which is critical for enhancing exercise capacity and overall physical activity levels in patients.

#### TEAEs

**Figure 9:**
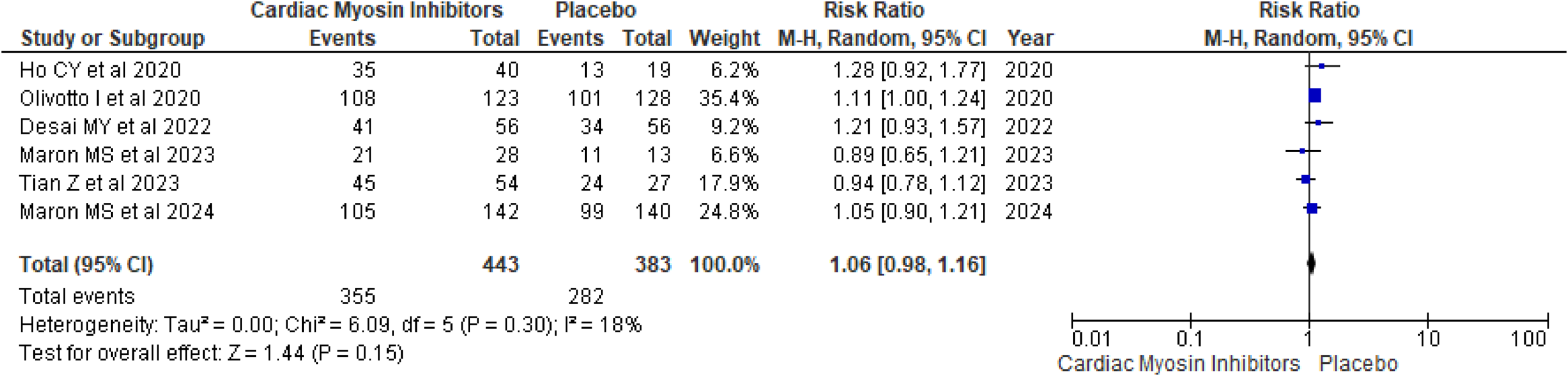
Forest plot for TEAEs.

The analysis of TEAEs yielded a RR of 1.06 (95% CI: 0.98, 1.16; *p* = 0.15; *I*^2^ = 18%; Figure 9), indicating no significant difference between the aficamten/mavacamten and control groups. This suggests that the treatment does not significantly increase the risk of adverse events, supporting its safety profile.

A drug-specific subgroup analysis revealed that both aficamten and mavacamten had a comparable number of TEAEs to placebo, with *p*-values of 0.81 and 0.15, respectively (see Supplementary Figure 8).

#### SAEs

**Figure 10:**
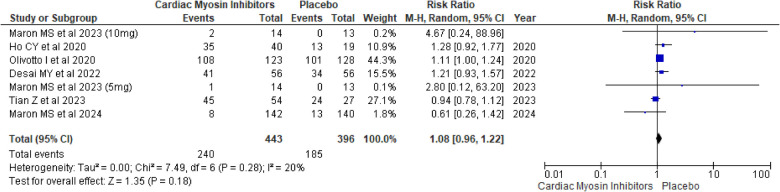
Forest plot for SAEs.

SAEs were also evaluated, showing a RR of 1.08 (95% CI: 0.96, 1.22; *p* = 0.18; *I*^2^ = 20%; Figure 10). The lack of significant difference between the groups indicates that the treatment does not lead to a higher incidence of serious adverse events, further reinforcing its safety.

A drug-specific subgroup analysis revealed that both aficamten and mavacamten had a comparable number of SAEs to placebo, with a *p*-value of 0.96 and 0.15 respectively (see Supplementary Figure 9).

## Discussion

In this systematic review and meta-analysis involving 826 patients with symptomatic HCM, we evaluated the efficacy and safety of cardiac myosin inhibitors compared to placebo. Our findings demonstrated substantial improvements in clinical outcomes, with significant enhancements in NYHA functional class and KCCQ-CSS. Both aficamten and mavacamten significantly reduced the mean Valsalva and resting LVOT peak gradients, suggesting effective reduction of LVOT obstruction severity both at rest and during exertion. Mavacamten also demonstrated significant reductions in post-exercise LVOT gradients, emphasizing its role in improving exercise capacity for patients. A modest decrease in LVEF was observed. The safety profiles of aficamten and mavacamten were comparable to placebo, with no significant difference in the incidence of TEAEs or SAEs between the intervention and control groups, supporting the safety of these treatments. Sensitivity analyses addressed sources of heterogeneity in these outcomes, enhancing confidence in the robustness of our findings.

Mavacamten, a novel cardiac myosin inhibitor, represents a pioneering, cardiac-specific, allosteric modulator designed to directly address the sarcomeric mutations characteristic of HCM. By increasing the population of myosin heads in the super-relaxed (SRX) state, Mavacamten shifts them toward ordered off-states near the thick filament backbone, thereby reducing the hypercontractile state that compromises myocardial compliance (24). It primarily targets the cardiac myosin heavy chain, which plays a key role in rendering the sarcomere susceptible to the deleterious, hypercontractile condition typical of HCM (25). Mavacamten reversibly affects several stages of the myosin chemomechanical cycle, reducing contractility and improving myocardial energetics. This includes partial inhibition of phosphatase release, decreased myosin S1 head interaction with actin, slowed myosin binding to actin in the ADP-bound state, and reduced ADP release from myosin S1 (26). Aficamten (CK-274), akin to mavacamten, diminishes myocardial contractility through selective allosteric binding to cardiac myosin, thereby attenuating actin-myosin cross-bridge formation within the myosin chemomechanical cycle (27). However, aficamten binds to a distinct cardiac site and is less extensively metabolized by the liver's cytochrome P450 system compared to mavacamten, resulting in fewer drug-drug interactions. Additionally, aficamten has been meticulously engineered to display a shallow dose-response curve, manifesting only minimal reductions in left ventricular ejection fraction with escalating doses. This characteristic suggests a broad therapeutic window and features a plasma half-life that allows for individualized dose modifications, potentially on a biweekly basis (11).

Individuals with HCM are predisposed to severe complications such as heart failure, myocardial ischemia, arrhythmias, and sudden cardiac death, with numerous studies linking the severity of LVOT obstruction to an increased risk of sudden cardiac death (28, 29). LVOT obstruction is characterized by a resting pressure gradient of 30 mmHg or greater between the left ventricle and the outflow tract (30). In HCM, outflow tract obstruction arises as the mitral valve's anterior leaflet moves anteriorly during systole and impinges upon the hypertrophied interventricular septum, intensifying the obstruction. This blockage can be exacerbated by a pressure gradient, influenced by contractility and loading conditions, which further displaces the mitral valve leaflet. This effect is particularly pronounced during a Valsalva maneuver, where reductions in left ventricular end-diastolic volume, preload, and cardiac output amplify the LVOT gradient and exacerbate the characteristic murmur of HCM (31, 32). The risk of heart failure is exacerbated, in part, by significant systolic anterior motion (SAM) of the mitral valve, which elevates filling pressures within the left ventricle (33). In asymptomatic individuals or those with mild heart failure symptoms, LVOT obstruction is a crucial predictor of cardiovascular mortality; however, in cases of severe heart failure, the NYHA functional class serves as a more precise prognostic indicator of mortality (29). Prolonged LVOT obstruction in moderate-to-severe HCM variants fosters cardiac remodeling, thereby increasing susceptibility to myocardial ischemia and ventricular arrhythmias—the predominant cause of sudden cardiac death in young, asymptomatic HCM patients (34). Additionally, supraventricular arrhythmias are prevalent, with 25%–30% of HCM patients developing atrial fibrillation—a consequence of LVOT obstruction and SAM of the mitral valve—which can lead to mitral regurgitation and resultant left atrial enlargement and dysfunction (35).

This meta-analysis demonstrated a significant improvement in the KCCQ-CSS, which assesses patient-reported symptoms, physical limitations, and quality of life, underscoring the positive impact of treatment on patients' perceived health status and daily functioning. In the EXPLORER-HCM trial, the Kansas City Cardiomyopathy Questionnaire (KCCQ) was validated for use in patients with obstructive HCM, showing that changes in KCCQ scores of 5, 10, and 20 points represent small, moderate, and large improvements, respectively, in symptoms, physical limitations, and quality of life. The improvement observed in our study (MD of 7.69) therefore aligns with clinically meaningful changes previously established for heart failure interventions, suggesting that the benefits of aficamten and mavacamten in HCM are both statistically and clinically significant (36). However, substantial heterogeneity was observed in this outcome, which decreased from 89% to 57% in sensitivity analysis when excluding the study by Tian et al. This reduction is likely due to the higher baseline KCCQ-CSS score of 82.4 in the Tian et al. study, which contributed to variability in treatment response. Additionally, both cardiac myosin inhibitors markedly diminished the mean peak gradients of LVOT during Valsalva and at rest. The observed attenuation in LVOT obstruction severity implies a potential corresponding decrease in the risk of several severe downstream consequences of HCM, such as heart failure, ventricular arrhythmias, cardiac remodeling, and sudden cardiac death.

This meta-analysis stands out as the first comprehensive evaluation of six recent RCTs examining the efficacy and safety of aficamten and mavacamten in treating symptomatic HCM. The rigorous subgroup and sensitivity analyses enhance the robustness of the findings, offering detailed insights into drug efficacy and safety. However, several limitations must be considered. The variability in follow-up periods across the studies could impact the assessment of primary and secondary outcomes. The heterogeneous designs and patient populations across the included studies pose challenges, with five RCTs focusing on oHCM and one on nHCM. Our inability to stratify data for obstructive and non-obstructive HCM separately due to insufficient data limits the generalizability of our results. This disparity highlights that the data supporting conclusions for obstructive HCM are more robust, whereas the evidence for non-obstructive HCM remains limited. Given the distinct pathophysiological and clinical characteristics of non-obstructive HCM, the applicability of our findings to this subgroup should be interpreted with caution. Further high-quality research is needed to explore therapeutic strategies specifically tailored to non-obstructive HCM. Another important limitation is the lack of data on the effects of mavacamten and aficamten on left ventricular diastolic function, an essential feature of both obstructive and non-obstructive HCM linked with myocardial fibrosis. Although these agents may potentially benefit diastolic function, current evidence does not adequately address this aspect. Additionally, patients receiving background therapy with beta-blockers and calcium channel blockers may have influenced the outcomes. Importantly, none of the included trials reported data on the discontinuation of medication due to decreased ejection fraction, a clinically significant outcome for myosin inhibitors, limiting the scope of our safety assessment. Furthermore, the current trials do not comprehensively evaluate the long-term effects of aficamten and mavacamten on clinical outcomes such as mortality and sudden cardiac death risk. Future research with extended follow-up periods is necessary to address these gaps.

In summary, this comprehensive meta-analysis evaluates the efficacy and safety of aficamten and mavacamten in treating patients with HCM. The findings reveal significant improvements in NYHA functional class and patient-reported outcomes as measured by the KCCQ-CSS, underscoring the effectiveness of cardiac myosin inhibitors in enhancing cardiac function and overall well-being. Both drugs notably reduced the mean Valsalva and rest LVOT peak gradients, indicating a substantial improvement in hemodynamic profiles. However, a modest decrease in LVEF was observed. Importantly, the treatments did not significantly increase the risk of adverse events or serious adverse events, supporting their safety. These results highlight the potential of aficamten and mavacamten as effective interventions for managing HCM, with further research needed to confirm long-term benefits and address any residual uncertainties.

## Data Availability

The original contributions presented in the study are included in the article/Supplementary Material, further inquiries can be directed to the corresponding author.
