# Supplementary material for: Efficacy and safety of cardiac myosin inhibitors for symptomatic hypertrophic cardiomyopathy: a meta-analysis of randomized controlled trials
Source: Front Cardiovasc Med. 2025 Jan 15;11:1477487. doi: 10.3389/fcvm.2024.1477487 (PMC11776027; doi:10.3389/fcvm.2024.1477487)

Supplementary Table 1. Risk of Bias Assessment Table

	Cochrane Risk-of-Bias Tool		
	Bias	Risk of bias	Author judgement
Desai MY et al<br>2022<br>NCT04349072	Random sequence generation (selection bias)	Low Risk	The study mentions that patients were randomized 1:1 to mavacamten or placebo, which implies a random sequence generation. However, specific details on how the randomization sequence was generated (e.g., computer-generated random numbers) are not provided.
	Allocation concealment (selection bias)	Low Risk	The study states that all study personnel remained blinded to treatment assignments until database lock, suggesting that allocation concealment was likely adequate.
	Blinding of participants and personnel (performance bias)	Low Risk	The trial was double-blind, meaning both participants and personnel were unaware of the treatment assignments, effectively minimizing performance bias.
	Blinding of outcome assessment (detection bias)	Low Risk	The core laboratory at the Cleveland Clinic, which was blinded to all study personnel, performed echocardiography assessments. This ensures that outcome assessment was not influenced by knowledge of treatment allocation.
	Incomplete outcome data (attrition bias)	Unclear Risk	The methods do not specify how incomplete outcome data were handled (e.g., whether intention-to-treat analysis was used for all outcomes). While the intention-to-treat population was mentioned for efficacy analyses, further details on handling dropouts and missing data are necessary for a conclusive assessment.
	Selective reporting (reporting bias)	Low Risk	The study protocol and statistical analysis plan are available in the Supplemental Appendix, indicating that predefined outcomes were likely reported as planned. There is no indication of selective reporting.
	Other bias	Low Risk	The trial was funded by MyoKardia, Inc., and coordinated by independent organizations (C5Research and Medpace), with academic oversight provided by an executive committee. The independent data monitoring committee had access to unblinded data, adding another layer of oversight. These factors reduce the likelihood of other biases.

Ho CY et al<br>2020<br>NCT03442764	Random sequence generation (selection bias)	Low Risk	The study states that participants were randomized 1:1:1 to different treatment groups, implying random sequence generation. However, specific details on the method used (e.g., computer-generated random numbers) are not provided.
	Allocation concealment (selection bias)	Low Risk	The study mentions blinded dose adjustments and the involvement of an interactive response system for handling treatment stop criteria, suggesting adequate allocation concealment.
	Blinding of participants and personnel (performance bias)	Low Risk	The trial was double-blind, with participants and personnel unaware of the treatment assignments, minimizing performance bias.
	Blinding of outcome assessment (detection bias)	Low Risk	Core laboratories were used for pharmacokinetic measurements and dose adjustments, and the protocol-defined treatment stop criteria were handled in a blinded environment, ensuring that outcome assessments were not influenced by knowledge of treatment allocation
	Incomplete outcome data (attrition bias)	Unclear Risk	The methods do not provide detailed information on how incomplete outcome data were handled (e.g., whether intention-to-treat analysis was used for all outcomes). While the efficacy analyses were performed on the intention-to-treat population, further details on handling dropouts and missing data are necessary for a conclusive assessment.
	Selective reporting (reporting bias)	Low Risk	The study protocol was approved by institutional review boards, and detailed safety and exploratory efficacy analyses were described. There is no indication of selective reporting.
	Other bias	Low Risk	the trial was conducted according to good clinical practice regulations and guidelines, with oversight by an independent Data Monitoring Committee, reducing the likelihood of other biases.
Maron MS et al<br>2023<br>NCT04219826	Random sequence generation (selection bias)	Low Risk	The study mentions that patients were randomized 2:1 to aficamten vs placebo. However, specific details on the method used for random sequence generation (e.g., computer-generated random numbers) are not provided.
	Allocation concealment (selection bias)	Low Risk	The study states that an interactive web response system was used for dispensing the proper dose based on echocardiographic criteria, ensuring that the allocation was concealed from the investigators.

	Blinding of participants and personnel (performance bias)	Low Risk	The trial was double-blind, with participants and personnel unaware of the treatment assignments, minimizing performance bias.
	Blinding of outcome assessment (detection bias)	Low Risk	Echocardiographic data were determined by a central core laboratory blinded to treatment assignment. Additionally, an unblinded sonographer performed the echocardiograms, and an unblinded cardiologist (not the study investigator) interpreted the results, ensuring outcome assessments were not influenced by knowledge of treatment allocation.
	Incomplete outcome data (attrition bias)	Unclear Risk	The methods do not provide detailed information on how incomplete outcome data were handled. While safety and exploratory efficacy analyses were performed, further details on handling dropouts and missing data are necessary for a conclusive assessment.
	Selective reporting (reporting bias)	Low Risk	The study protocol was approved by institutional review boards, and detailed safety and exploratory efficacy analyses were described. There is no indication of selective reporting.
	Other bias	Low Risk	The trial was conducted according to good clinical practice regulations and guidelines, with oversight by an independent Data Monitoring Committee, reducing the likelihood of other biases.
Maron MS et al 2024 NCT05186818	Random sequence generation (selection bias)	Low Risk	The study used an interactive web-response system for randomization, which suggests a systematic and unbiased method for sequence generation.
	Allocation concealment (selection bias)	Low Risk	The interactive web-response system also ensured allocation concealment by keeping the randomization process blind to the investigators and participants.
	Blinding of participants and personnel (performance bias)	Low Risk	The trial was double-blind, with both participants and personnel unaware of the treatment assignments, minimizing performance bias.
	Blinding of outcome assessment (detection bias)	Low Risk	Echocardiographic and cardiopulmonary exercise testing data were analyzed by a core laboratory blind to the treatment groups. Additionally, site investigators and trial team members were unaware of NT-proBNP levels and echocardiography results, ensuring unbiased outcome assessment.

	Incomplete outcome data (attrition bias)	Unclear Risk	While the study mentions the use of multiple imputation for missing data, further details on handling incomplete outcome data and reasons for missing data are not fully provided. This makes it difficult to assess the impact of attrition bias conclusively.
	Selective reporting (reporting bias)	Low Risk	The study protocol and statistical analysis plan are available, and the trial design, including primary and secondary endpoints, is clearly described. There is no indication of selective reporting.
	Other bias	Low Risk	The trial was conducted according to good clinical practice guidelines, with oversight by an independent data monitoring committee, reducing the likelihood of other biases.
Olivotto I et al<br>2020<br>NCT03470545	Random sequence generation (selection bias)	Low Risk	The randomization was performed using an interactive response system, ensuring a systematic and unbiased generation of random sequences.
	Allocation concealment (selection bias)	Low Risk	Allocation concealment was maintained by using the interactive response system, which kept the treatment assignments hidden from both investigators and participants.
	Blinding of participants and personnel (performance bias)	Low Risk	The trial was double-blind, and both the investigators and participants, as well as the central and core laboratories and clinical site monitors, were masked to the treatment assignments.
	Blinding of outcome assessment (detection bias)	Low Risk	Outcome assessments, including echocardiographic and CPET data, were conducted by central core laboratories that were masked to treatment assignments.
	Incomplete outcome data (attrition bias)	Low Risk	The trial used predefined methods for handling missing data, including imputation strategies for specific outcomes. Missing data were not imputed unless specified, and the analyses followed the intention-to-treat principle.
	Selective reporting (reporting bias)	Low Risk	The trial protocol and statistical analysis plan were predefined and followed, with primary and secondary endpoints clearly outlined. There is no indication of selective reporting.
	Other bias	Low Risk	The trial was conducted in accordance with good clinical practice guidelines and was overseen by independent committees, reducing the likelihood of other biases.
Tian Z et al<br>2023	Random sequence generation (selection bias)		Patients were randomized using an interactive response system, ensuring randomization was systematic and unbiased.

NCT05174416			
	Allocation concealment (selection bias)		Allocation concealment was maintained through the interactive response system, keeping treatment assignments hidden from investigators and participants.
	Blinding of participants and personnel (performance bias)		The trial was double-blind, and all relevant parties including patients, study investigators, staff, the sponsor, and central/core laboratories were masked to treatment assignment. The placebo and active drug were identical in appearance, supporting effective blinding.
	Blinding of outcome assessment (detection bias)		Outcomes were assessed by core laboratories and using methods such as echocardiography and CMR imaging, which were masked to treatment assignments. This minimizes detection bias.
	Incomplete outcome data (attrition bias)		The study planned for a dropout rate of 10% and accounted for this in the sample size calculation. Missing data were handled according to the intention-to-treat principle, and CMR imaging outcomes were based on available data.
	Selective reporting (reporting bias)		The trial adhered to the predefined statistical analysis plan, and the protocol outlined primary, secondary, and exploratory endpoints. All outcomes reported were part of the planned analysis.
	Other bias		The study followed rigorous guidelines including the Declaration of Helsinki and Good Clinical Practice. It was approved by regulatory authorities and independent ethics committees, and the trial was registered with ClinicalTrials.gov.

Supplementary Figure 1: Forest plot for subgroup analysis of Improvement in NYHA Functional Class

Study or Subgroup	Cardiac Myosin Inhibitors Events	Cardiac Myosin Inhibitors Total	Placebo Events	Placebo Total	Weight	Risk Ratio M-H, Random, 95% CI	Year
**1.9.1 Aficamten**							
Maron MS et al 2023 (10mg)	9	14	4	13	5.4%	2.09 [0.85, 5.16]	
Maron MS et al 2023 (5mg)	6	14	4	13	4.4%	1.39 [0.50, 3.84]	2023
Maron MS et al 2024	83	142	34	140	29.0%	2.41 [1.74, 3.33]	2024
**Subtotal (95% CI)**		**170**		**166**	**38.9%**	**2.27 [1.69, 3.04]**	
Total events	98		42				
Heterogeneity: Tau² = 0.00; Chi² = 1.05, df = 2 (P = 0.59); I² = 0%							
Test for overall effect: Z = 5.50 (P < 0.00001)							
**1.9.2 Mavacamten**							
Olivotto I et al 2020	80	123	40	128	33.5%	2.08 [1.56, 2.78]	2020
Ho CY et al 2020	17	40	7	19	8.9%	1.15 [0.58, 2.30]	2020
Desai MY et al 2022	35	56	12	56	13.6%	2.92 [1.70, 5.01]	2022
Tian Z et al 2023	32	54	4	27	5.2%	4.00 [1.58, 10.15]	2023
**Subtotal (95% CI)**		**273**		**230**	**61.1%**	**2.21 [1.48, 3.30]**	
Total events	164		63				
Heterogeneity: Tau² = 0.08; Chi² = 6.24, df = 3 (P = 0.10); I² = 52%							
Test for overall effect: Z = 3.85 (P = 0.0001)							
**Total (95% CI)**		**443**		**396**	**100.0%**	**2.19 [1.76, 2.73]**	
Total events	262		105				
Heterogeneity: Tau² = 0.02; Chi² = 7.32, df = 6 (P = 0.29); I² = 18%							
Test for overall effect: Z = 7.04 (P < 0.00001)							
Test for subgroup differences: Chi² = 0.01, df = 1 (P = 0.91), I² = 0%							

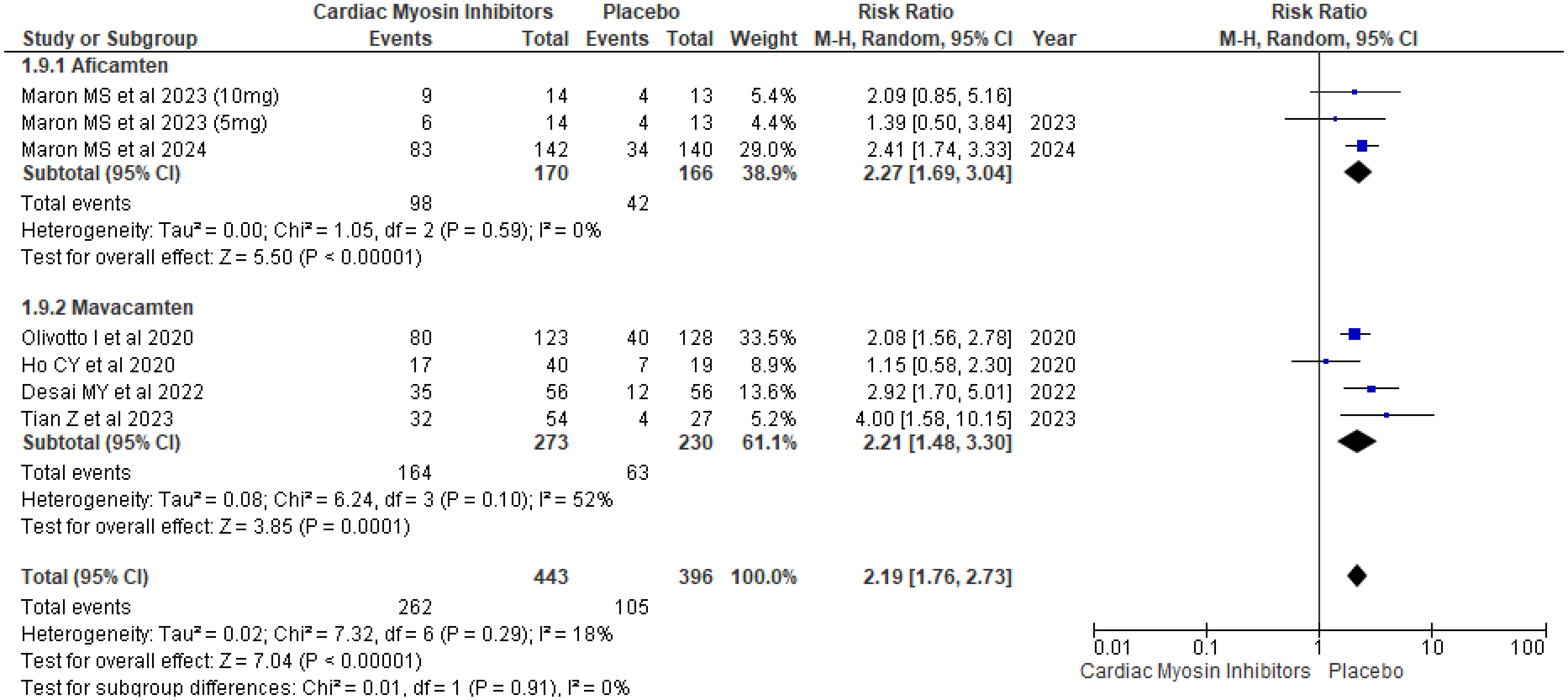

Supplementary Figure 2: Forest plot for subgroup analysis of Improvement in KCCQ-CSS

Study or Subgroup	Cardiac Myosin Inhibitor Mean	Cardiac Myosin Inhibitor SD	Cardiac Myosin Inhibitor Total	Placebo Mean	Placebo SD	Placebo Total	Weight	Mean Difference IV, Random, 95% CI	Year
**1.10.2 Aficamten**									
Maron MS et al 2024	11.3	3.7	142	5	2.96	140	28.7%	6.30 [5.52, 7.08]	2024
**Subtotal (95% CI)**			**142**			**140**	**28.7%**	**6.30 [5.52, 7.08]**	
Heterogeneity: Not applicable									
Test for overall effect: Z = 15.80 (P < 0.00001)									
**1.10.3 Mavacamten**									
Ho CY et al 2020	3.37	9.41	40	4.34	16.05	19	8.9%	-0.97 [-8.75, 6.81]	2020
Olivotto I et al 2020	13.6	14.4	123	4.2	13.7	128	20.1%	9.40 [5.92, 12.88]	2020
Desai MY et al 2022	10.4	16.1	56	1.9	12	56	14.3%	8.50 [3.24, 13.76]	2022
Tian Z et al 2023	4.99	2.06	54	-5.25	2.75	27	27.9%	10.24 [9.07, 11.41]	2023
**Subtotal (95% CI)**			**273**			**230**	**71.3%**	**8.38 [5.31, 11.45]**	
Heterogeneity: Tau² = 5.61; Chi² = 8.16, df = 3 (P = 0.04); I² = 63%									
Test for overall effect: Z = 5.34 (P < 0.00001)									
**Total (95% CI)**			**415**			**370**	**100.0%**	**7.69 [4.91, 10.47]**	
Heterogeneity: Tau² = 6.84; Chi² = 35.81, df = 4 (P < 0.00001); I² = 89%									
Test for overall effect: Z = 5.43 (P < 0.00001)									
Test for subgroup differences: Chi² = 1.65, df = 1 (P = 0.20), I² = 39.5%									

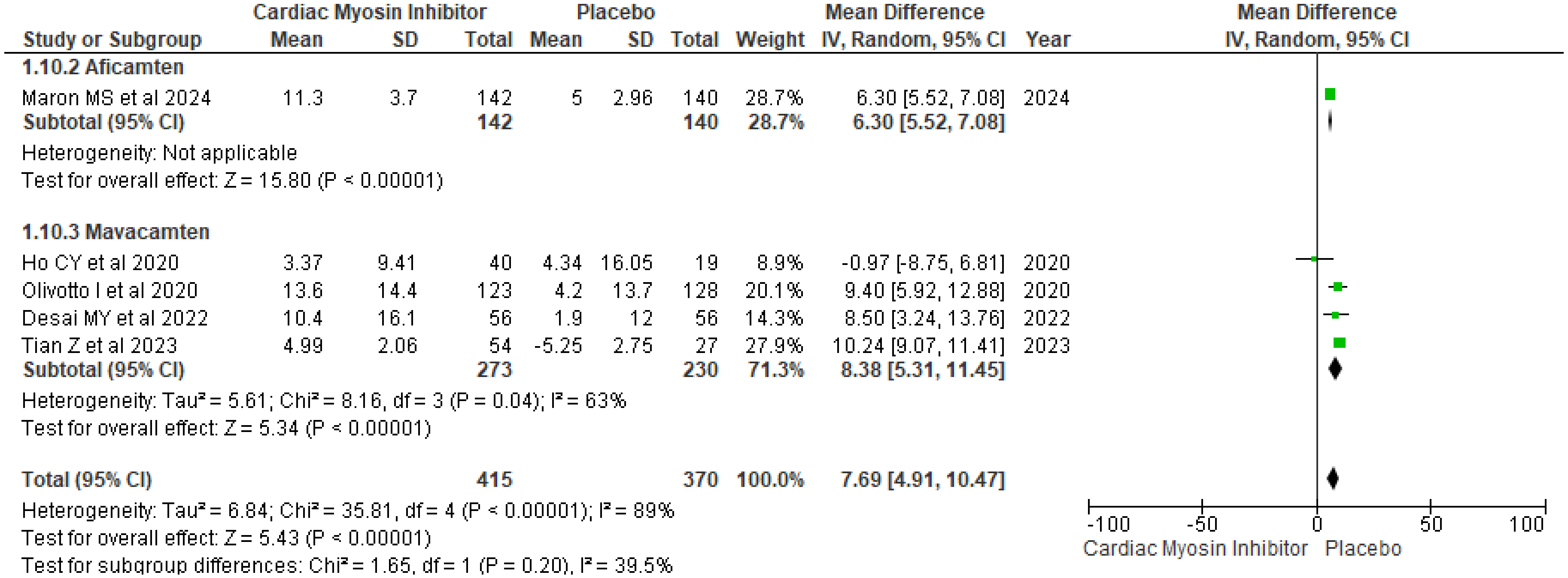

Supplementary Figure 3: Forest plot for sensitivity analysis for Improvement in KCCQ-CSS

Study or Subgroup	Cardiac Myosin Inhibitor Mean	SD	Total	Placebo Mean	SD	Total	Weight	Mean Difference IV, Random, 95% CI	Year
**1.10.2 Aficamten**									
Maron MS et al 2024	11.3	3.7	142	5	2.96	140	46.1%	6.30 [5.52, 7.08]	2024
**Subtotal (95% CI)**			**142**			**140**	**46.1%**	**6.30 [5.52, 7.08]**	
Heterogeneity: Not applicable									
Test for overall effect: Z = 15.80 (P < 0.00001)									
**1.10.3 Mavacamten**									
Ho CY et al 2020	3.37	9.41	40	4.34	16.05	19	9.8%	-0.97 [-8.75, 6.81]	2020
Olivotto I et al 2020	13.6	14.4	123	4.2	13.7	128	26.9%	9.40 [5.92, 12.88]	2020
Desai MY et al 2022	10.4	16.1	56	1.9	12	56	17.2%	8.50 [3.24, 13.76]	2022
Tian Z et al 2023	4.99	2.06	54	-5.25	2.75	27	0.0%	10.24 [9.07, 11.41]	2023
**Subtotal (95% CI)**			**219**			**203**	**53.9%**	**6.62 [1.44, 11.79]**	
Heterogeneity: Tau² = 13.36; Chi² = 5.75, df = 2 (P = 0.06); I² = 65%									
Test for overall effect: Z = 2.51 (P = 0.01)									
**Total (95% CI)**			**361**			**343**	**100.0%**	**6.80 [4.07, 9.53]**	
Heterogeneity: Tau² = 4.05; Chi² = 6.97, df = 3 (P = 0.07); I² = 57%									
Test for overall effect: Z = 4.89 (P < 0.00001)									
Test for subgroup differences: Chi² = 0.01, df = 1 (P = 0.91), I² = 0%									

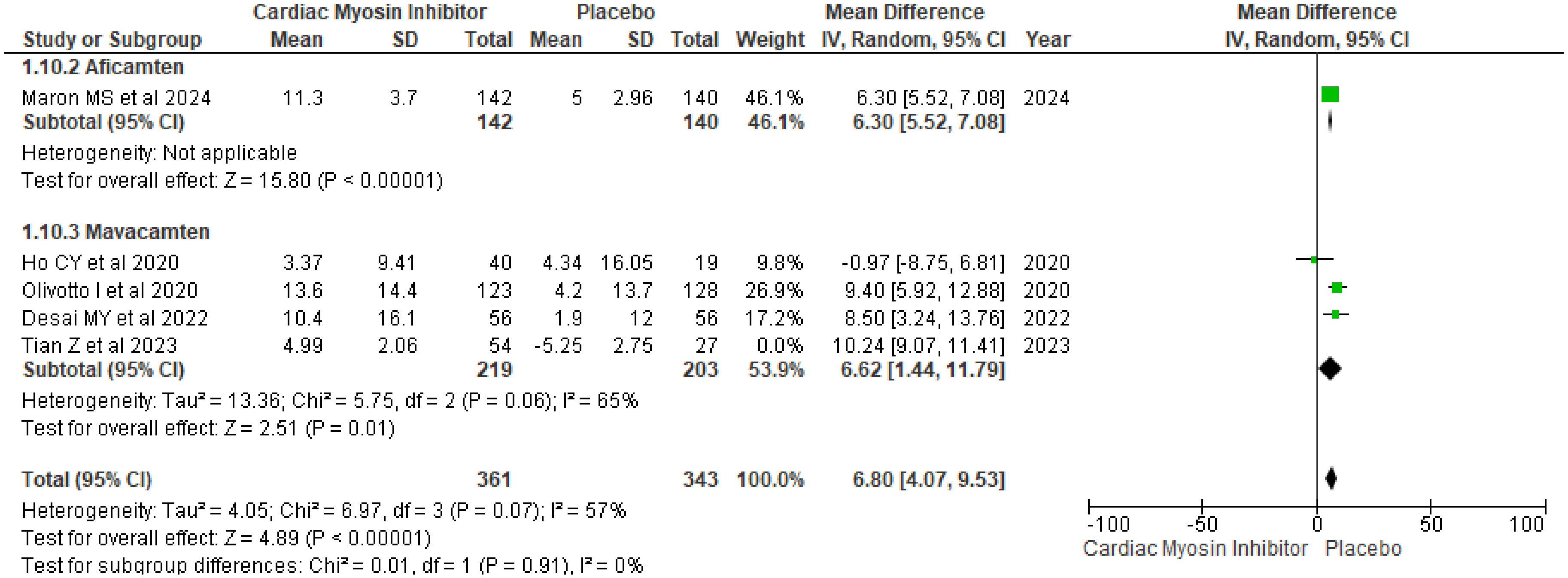

Supplementary Figure 4: Forest plot for subgroup analysis of Mean Valsalva LVOT Peak Gradient

Study or Subgroup	Cardiac Myosin Inhibitors Mean	SD	Total	Placebo Mean	SD	Total	Weight	Mean Difference IV, Random, 95% CI	Year
**1.13.2 Aficamten**									
Maron MS et al 2023 (10mg)	-43	34.0491	14	-8	24.1572	13	15.8%	-35.00 [-57.15, -12.85]	
Maron MS et al 2023 (5mg)	-33	34.4232	14	-8	24.1572	13	15.7%	-25.00 [-47.31, -2.69]	2023
Maron MS et al 2024	-47.6	39.7829	142	1.8	34.7093	140	26.9%	-49.40 [-58.11, -40.69]	2024
**Subtotal (95% CI)**			**170**			**166**	**58.4%**	**-39.28 [-54.79, -23.77]**	
Heterogeneity: Tau² = 111.23; Chi² = 4.85, df = 2 (P = 0.09); I² = 59%									
Test for overall effect: Z = 4.96 (P < 0.00001)									
**1.13.3 Mavacamten**									
Desai MY et al 2022	-45.2	28.8	56	0.4	29.7	56	25.2%	-45.60 [-56.44, -34.76]	2022
Tian Z et al 2023	-57.93	45.614	54	20.65	46.448	27	16.4%	-78.58 [-99.91, -57.25]	2023
**Subtotal (95% CI)**			**110**			**83**	**41.6%**	**-60.76 [-92.97, -28.54]**	
Heterogeneity: Tau² = 469.34; Chi² = 7.30, df = 1 (P = 0.007); I² = 86%									
Test for overall effect: Z = 3.70 (P = 0.0002)									
**Total (95% CI)**			**280**			**249**	**100.0%**	**-47.12 [-59.72, -34.52]**	
Heterogeneity: Tau² = 133.66; Chi² = 13.58, df = 4 (P = 0.009); I² = 71%									
Test for overall effect: Z = 7.33 (P < 0.00001)									
Test for subgroup differences: Chi² = 1.39, df = 1 (P = 0.24), I² = 27.9%									

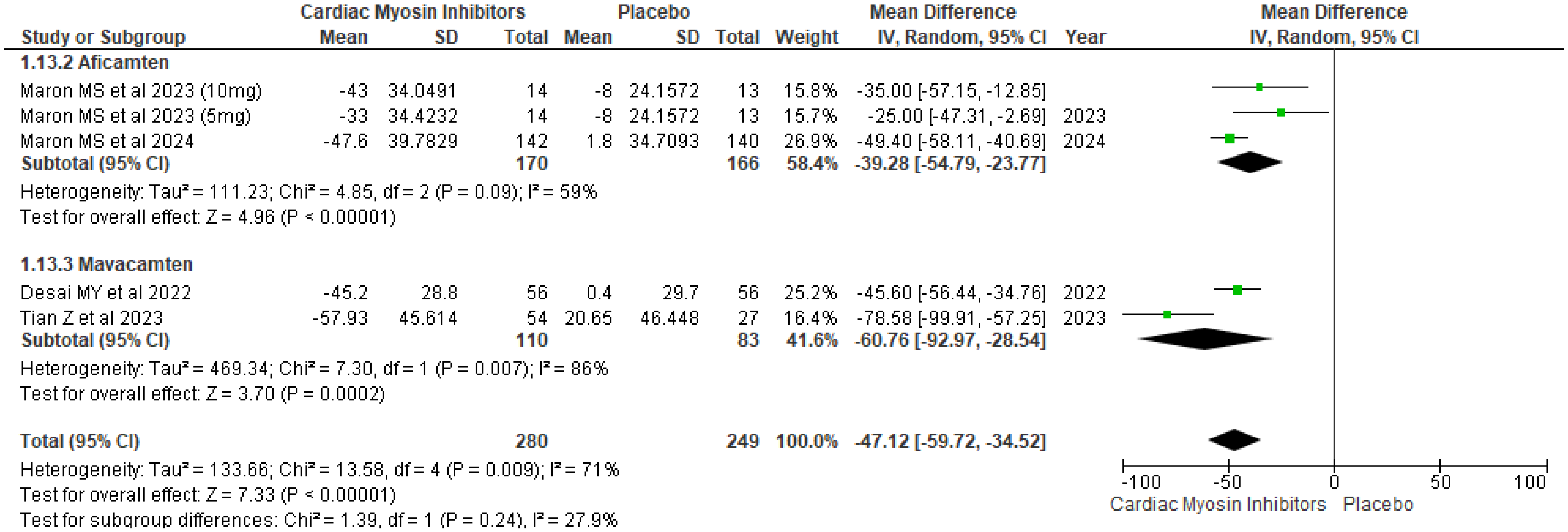

Supplementary Figure 5: Forest plot for sensitivity analysis for Mean Valsalva LVOT Peak Gradient

Study or Subgroup	Cardiac Myosin Inhibitors Mean	SD	Total	Placebo Mean	SD	Total	Weight	Mean Difference IV, Random, 95% CI	Year
**1.13.2 Aficamten**									
Maron MS et al 2023 (10mg)	-43	34.0491	14	-8	24.1572	13	12.9%	-35.00 [-57.15, -12.85]	
Maron MS et al 2023 (5mg)	-33	34.4232	14	-8	24.1572	13	12.8%	-25.00 [-47.31, -2.69]	2023
Maron MS et al 2024	-47.6	39.7829	142	1.8	34.7093	140	40.7%	-49.40 [-58.11, -40.69]	2024
**Subtotal (95% CI)**			**170**			**166**	**66.5%**	**-39.28 [-54.79, -23.77]**	
Heterogeneity: Tau² = 111.23; Chi² = 4.85, df = 2 (P = 0.09); I² = 59%									
Test for overall effect: Z = 4.96 (P < 0.00001)									
**1.13.3 Mavacamten**									
Desai MY et al 2022	-45.2	28.8	56	0.4	29.7	56	33.5%	-45.60 [-56.44, -34.76]	2022
Tian Z et al 2023	-57.93	45.614	54	20.65	46.448	27	0.0%	-78.58 [-99.91, -57.25]	2023
**Subtotal (95% CI)**			**56**			**56**	**33.5%**	**-45.60 [-56.44, -34.76]**	
Heterogeneity: Not applicable									
Test for overall effect: Z = 8.25 (P < 0.00001)									
**Total (95% CI)**			**226**			**222**	**100.0%**	**-43.14 [-52.01, -34.27]**	
Heterogeneity: Tau² = 30.56; Chi² = 4.86, df = 3 (P = 0.18); I² = 38%									
Test for overall effect: Z = 9.53 (P < 0.00001)									
Test for subgroup differences: Chi² = 0.43, df = 1 (P = 0.51), I² = 0%									

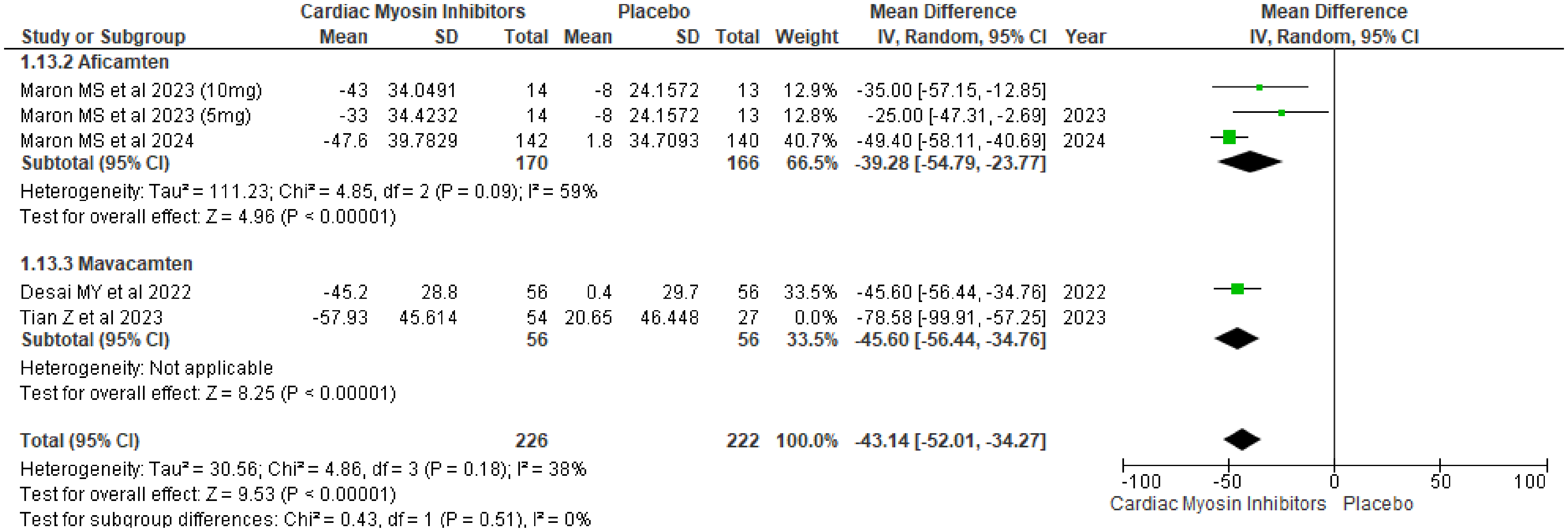

Supplementary Figure 6: Forest plot for subgroup analysis of Mean Rest LVOT Peak Gradient

Study or Subgroup	Cardiac Myosin Inhibitors Mean	SD	Total	Placebo Mean	SD	Total	Weight	Mean Difference IV, Random, 95% CI	Year
**1.14.2 Aficamten**									
Maron MS et al 2023 (10mg)	-28	26.9399	14	-13	18.7489	13	23.8%	-15.00 [-32.41, 2.41]	
Maron MS et al 2023 (5mg)	-29	26.9399	14	-13	18.7489	13	23.8%	-16.00 [-33.41, 1.41]	2023
**Subtotal (95% CI)**			**28**			**26**	**47.5%**	**-15.50 [-27.81, -3.19]**	
Heterogeneity: Tau² = 0.00; Chi² = 0.01, df = 1 (P = 0.94); I² = 0%									
Test for overall effect: Z = 2.47 (P = 0.01)									
**1.14.3 Mavacamten**									
Desai MY et al 2022	-36	28.8	56	-1.5	26.5	56	27.9%	-34.50 [-44.75, -24.25]	2022
Tian Z et al 2023	-51.45	35.985	54	6.38	34.356	27	24.6%	-57.83 [-73.96, -41.70]	2023
**Subtotal (95% CI)**			**110**			**83**	**52.5%**	**-45.30 [-68.10, -22.50]**	
Heterogeneity: Tau² = 224.62; Chi² = 5.73, df = 1 (P = 0.02); I² = 83%									
Test for overall effect: Z = 3.89 (P < 0.0001)									
**Total (95% CI)**			**138**			**109**	**100.0%**	**-31.20 [-48.97, -13.42]**	
Heterogeneity: Tau² = 267.22; Chi² = 16.95, df = 3 (P = 0.0007); I² = 82%									
Test for overall effect: Z = 3.44 (P = 0.0006)									
Test for subgroup differences: Chi² = 5.08, df = 1 (P = 0.02), I² = 80.3%									

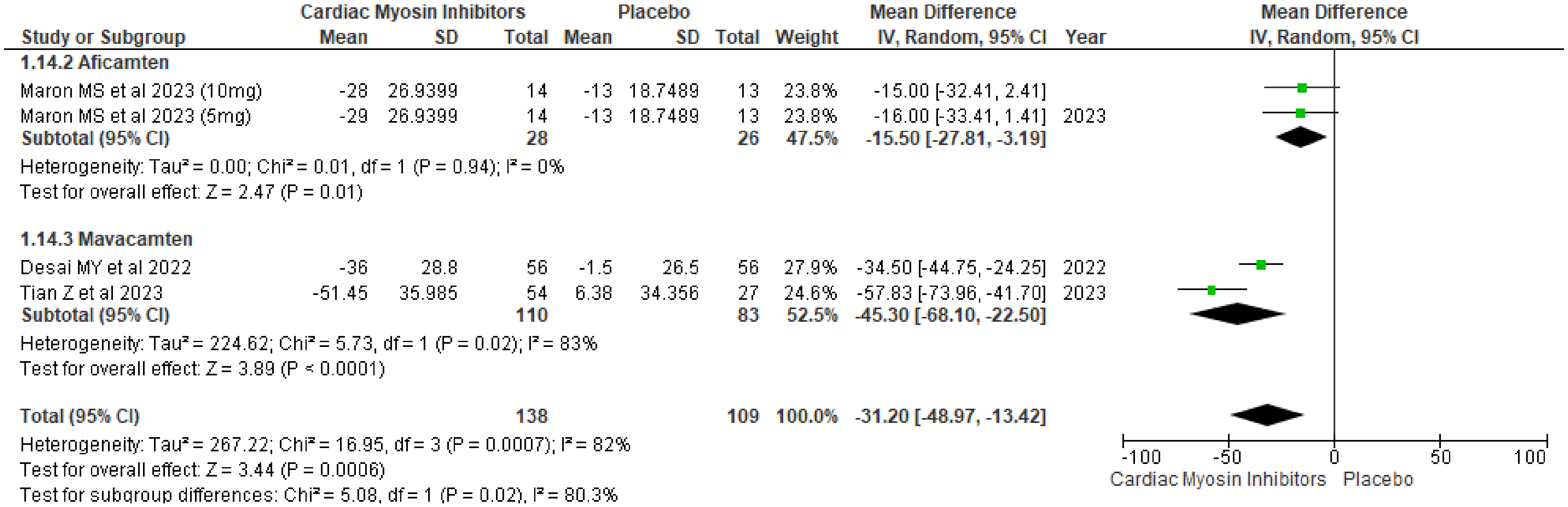

Supplementary Figure 7: Forest plot for sensitivity analysis for Mean Rest LVOT Peak Gradient

Study or Subgroup	Cardiac Myosin Inhibitors Mean	SD	Total	Placebo Mean	SD	Total	Weight	Mean Difference IV, Random, 95% CI	Year
**1.14.2 Aficamten**									
Maron MS et al 2023 (10mg)	-28	26.9399	14	-13	18.7489	13	29.3%	-15.00 [-32.41, 2.41]	
Maron MS et al 2023 (5mg)	-29	26.9399	14	-13	18.7489	13	29.3%	-16.00 [-33.41, 1.41]	2023
**Subtotal (95% CI)**			**28**			**26**	**58.6%**	**-15.50 [-27.81, -3.19]**	
Heterogeneity: Tau² = 0.00; Chi² = 0.01, df = 1 (P = 0.94); I² = 0%									
Test for overall effect: Z = 2.47 (P = 0.01)									
**1.14.3 Mavacamten**									
Desai MY et al 2022	-36	28.8	56	-1.5	26.5	56	41.4%	-34.50 [-44.75, -24.25]	2022
Tian Z et al 2023	-51.45	35.985	54	6.38	34.356	27	0.0%	-57.83 [-73.96, -41.70]	2023
**Subtotal (95% CI)**			**56**			**56**	**41.4%**	**-34.50 [-44.75, -24.25]**	
Heterogeneity: Not applicable									
Test for overall effect: Z = 6.60 (P < 0.00001)									
**Total (95% CI)**			**84**			**82**	**100.0%**	**-23.37 [-37.44, -9.30]**	
Heterogeneity: Tau² = 97.09; Chi² = 5.41, df = 2 (P = 0.07); I² = 63%									
Test for overall effect: Z = 3.26 (P = 0.001)									
Test for subgroup differences: Chi² = 5.40, df = 1 (P = 0.02), I² = 81.5%									

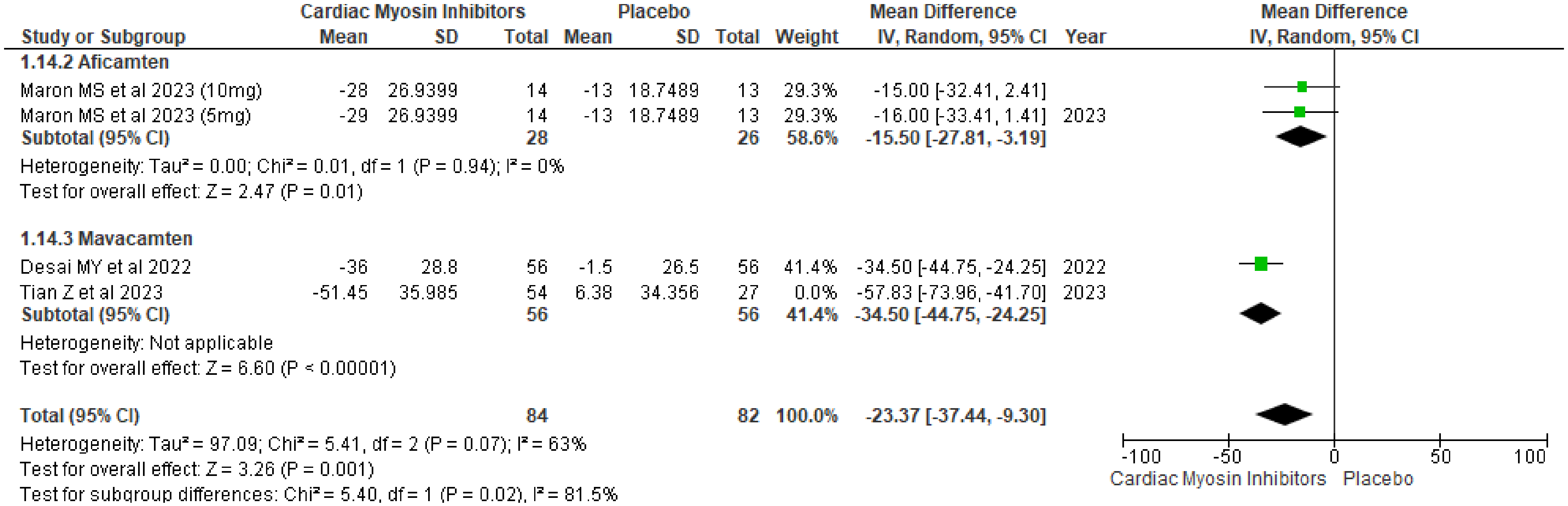

Supplementary Figure 8: Forest plot for subgroup analysis of TEAEs

Study or Subgroup	Cardiac Myosin Inhibitors Events	Total	Placebo Events	Total	Weight	Risk Ratio M-H, Random, 95% CI	Year
**1.11.2 Aficamten**							
Maron MS et al 2023	21	28	11	13	6.6%	0.89 [0.65, 1.21]	2023
Maron MS et al 2024	105	142	99	140	24.8%	1.05 [0.90, 1.21]	2024
**Subtotal (95% CI)**		**170**		**153**	**31.4%**	**1.02 [0.89, 1.16]**	
Total events	126		110				
Heterogeneity: Tau² = 0.00; Chi² = 0.89, df = 1 (P = 0.35); I² = 0%							
Test for overall effect: Z = 0.24 (P = 0.81)							
**1.11.3 Mavacamten**							
Ho CY et al 2020	35	40	13	19	6.2%	1.28 [0.92, 1.77]	2020
Olivotto I et al 2020	108	123	101	128	35.4%	1.11 [1.00, 1.24]	2020
Desai MY et al 2022	41	56	34	56	9.2%	1.21 [0.93, 1.57]	2022
Tian Z et al 2023	45	54	24	27	17.9%	0.94 [0.78, 1.12]	2023
**Subtotal (95% CI)**		**273**		**230**	**68.6%**	**1.09 [0.97, 1.23]**	
Total events	229		172				
Heterogeneity: Tau² = 0.01; Chi² = 4.63, df = 3 (P = 0.20); I² = 35%							
Test for overall effect: Z = 1.43 (P = 0.15)							
**Total (95% CI)**		**443**		**383**	**100.0%**	**1.06 [0.98, 1.16]**	
Total events	355		282				
Heterogeneity: Tau² = 0.00; Chi² = 6.09, df = 5 (P = 0.30); I² = 18%							
Test for overall effect: Z = 1.44 (P = 0.15)							
Test for subgroup differences: Chi² = 0.63, df = 1 (P = 0.43), I² = 0%							

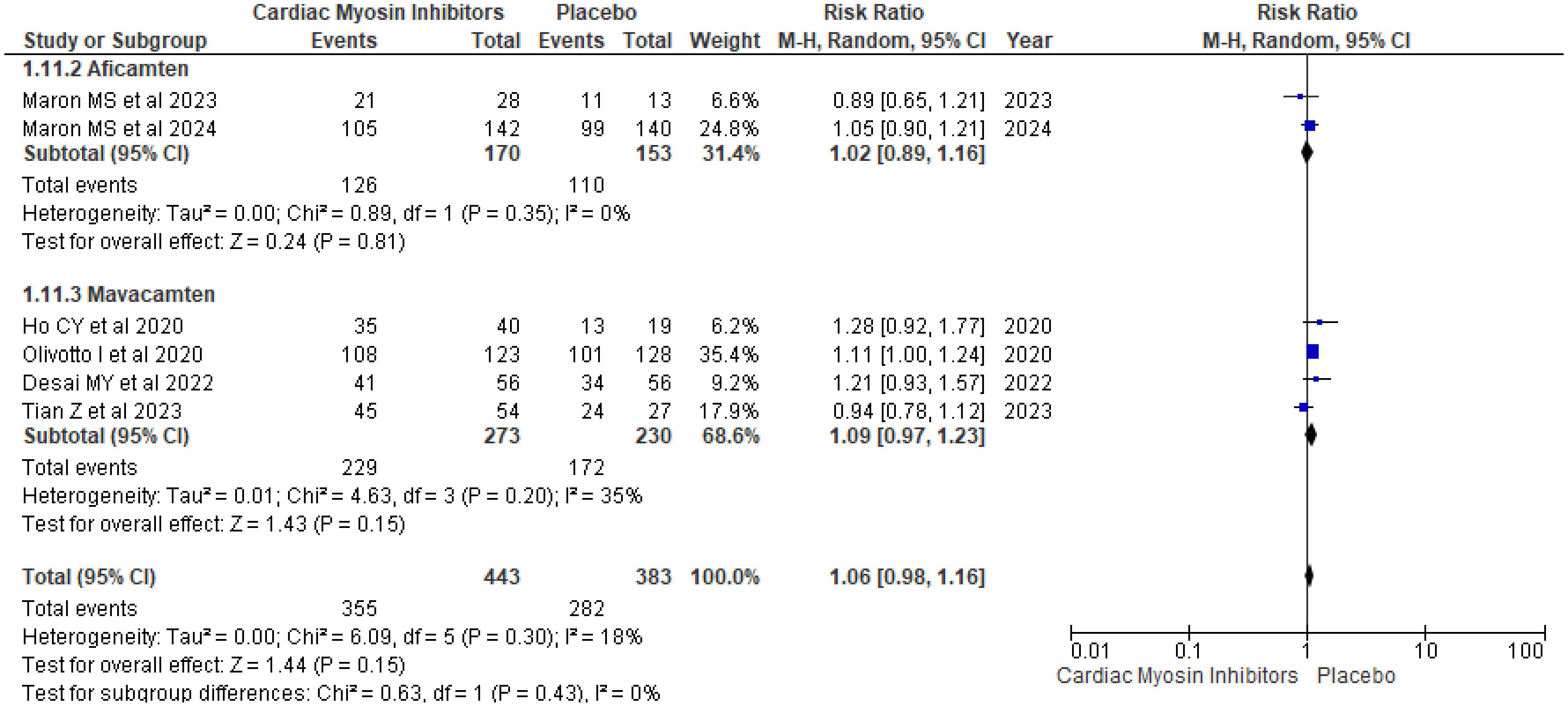

Supplementary Figure 9: Forest plot for subgroup analysis of SAEs

Study or Subgroup	Cardiac Myosin Inhibitors Events	Cardiac Myosin Inhibitors Total	Placebo Events	Placebo Total	Weight	Risk Ratio M-H, Random, 95% CI	Year
**1.12.2 Aficamten**							
Maron MS et al 2023 (10mg)	2	14	0	13	0.2%	4.67 [0.24, 88.96]	
Maron MS et al 2023 (5mg)	1	14	0	13	0.1%	2.80 [0.12, 63.20]	2023
Maron MS et al 2024	8	142	13	140	1.8%	0.61 [0.26, 1.42]	2024
**Subtotal (95% CI)**		**170**		**166**	**2.1%**	**0.97 [0.30, 3.10]**	
Total events	11		13				
Heterogeneity: Tau² = 0.29; Chi² = 2.43, df = 2 (P = 0.30); I² = 18%							
Test for overall effect: Z = 0.05 (P = 0.96)							
**1.12.3 Mavacamten**							
Olivotto I et al 2020	108	123	101	128	44.3%	1.11 [1.00, 1.24]	2020
Ho CY et al 2020	35	40	13	19	10.9%	1.28 [0.92, 1.77]	2020
Desai MY et al 2022	41	56	34	56	15.5%	1.21 [0.93, 1.57]	2022
Tian Z et al 2023	45	54	24	27	27.1%	0.94 [0.78, 1.12]	2023
**Subtotal (95% CI)**		**273**		**230**	**97.9%**	**1.09 [0.97, 1.23]**	
Total events	229		172				
Heterogeneity: Tau² = 0.01; Chi² = 4.63, df = 3 (P = 0.20); I² = 35%							
Test for overall effect: Z = 1.43 (P = 0.15)							
**Total (95% CI)**		**443**		**396**	**100.0%**	**1.08 [0.96, 1.22]**	
Total events	240		185				
Heterogeneity: Tau² = 0.00; Chi² = 7.49, df = 6 (P = 0.28); I² = 20%							
Test for overall effect: Z = 1.35 (P = 0.18)							
Test for subgroup differences: Chi² = 0.04, df = 1 (P = 0.84), I² = 0%							

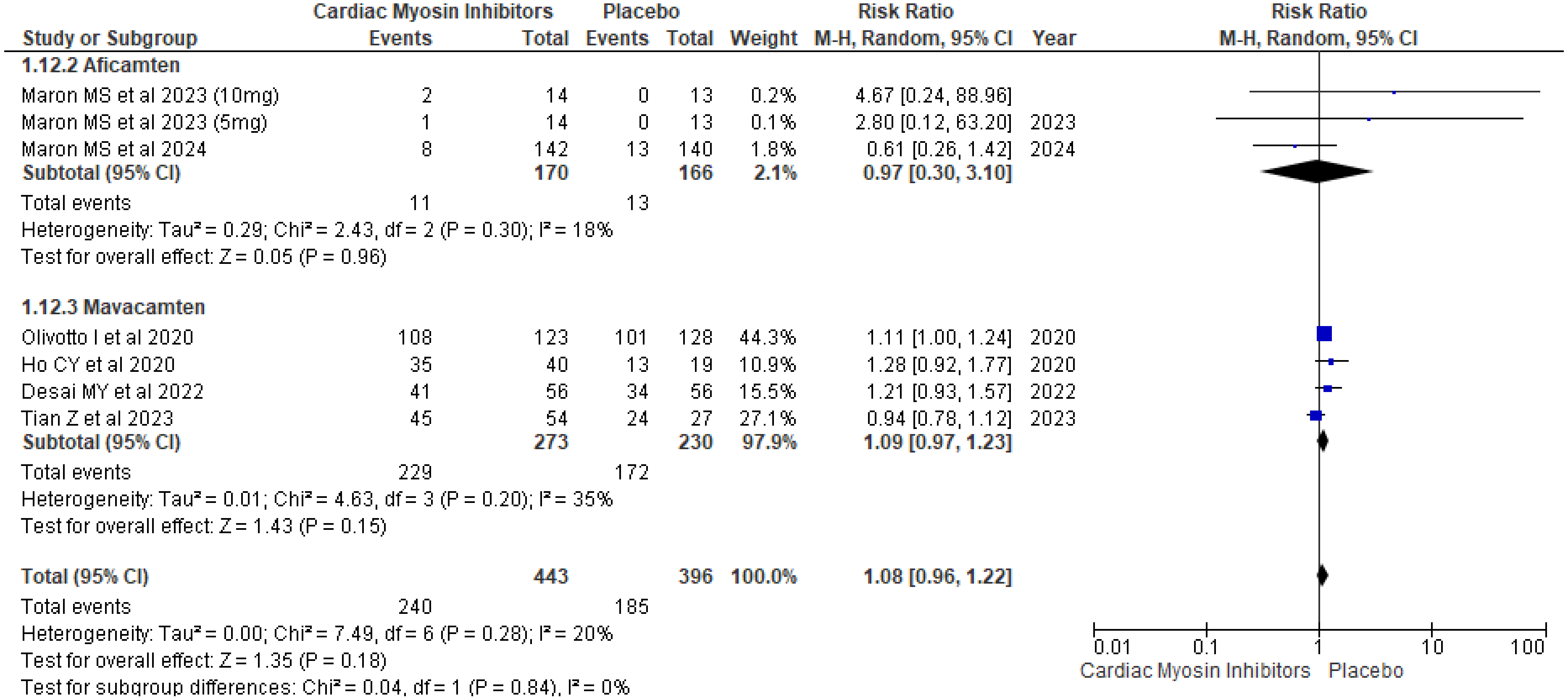

Supplement: Supplementary file 1 [file Datasheet1.pdf]
